# Fluoroscopy-guided simultaneous distal perfusion as a preventive strategy of limb ischemia in patients undergoing extracorporeal membrane oxygenation

**DOI:** 10.1186/s13613-018-0445-z

**Published:** 2018-10-29

**Authors:** Woo Jin Jang, Yang Hyun Cho, Taek Kyu Park, Young Bin Song, Jin-Oh Choi, Joo-Yong Hahn, Seung-Hyuk Choi, Hyeon-Cheol Gwon, Eun-Seok Jeon, Woo Jung Chun, Ju Hyeon Oh, Jeong Hoon Yang

**Affiliations:** 1 Division of Cardiology, Department of Internal Medicine, Samsung Changwon Hospital, Sungkyunkwan University School of Medicine, Changwon, Republic of Korea; 2 Department of Thoracic and Cardiovascular Surgery, Samsung Medical Center, Sungkyunkwan University School of Medicine, Seoul, Republic of Korea; 3 Division of Cardiology, Department of Critical Care Medicine and Medicine, Heart Vascular Stroke Institute, Samsung Medical Center, Sungkyunkwan University School of Medicine, 81, Irwon-dong, Gangnam-gu, Seoul, 135-710 Republic of Korea

**Keywords:** Distal perfusion catheter, Limb ischemia, Extracorporeal membrane oxygenation

## Abstract

**Background:**

Limited data are available regarding prevention of limb ischemia in femorally cannulated patients on venoarterial extracorporeal membrane oxygenation (VA-ECMO). We investigated the association between strategy of distal perfusion catheter (DPC) insertion and vascular complications like limb ischemia in patients undergoing VA-ECMO.

**Methods:**

We evaluated 230 patients from two tertiary hospitals who received VA-ECMO via femoral cannulation between August 2014 and July 2017. The patients were divided into two groups according to DPC insertion strategy: patients who underwent DPC insertion at the time of primary cannulation (DPC group, *n* = 96) and patients who were provisionally treated with DPC (No-DPC group, *n* = 134). The primary outcome was limb ischemia.

**Results:**

Of the 96 patients in the DPC group, 61 (63.5%) underwent insertion under fluoroscopic guidance. The DPC group had a significantly lower incidence of limb ischemia (2.1% vs. 8.2%, *p* = 0.047) and a lower tendency of in-hospital mortality (38.5% vs. 50.7%, *p* = 0.067) than the No-DPC group. In the multivariable analysis, fluoroscopy-guided simultaneous insertion of the DPC (odds ratio 0.11; 95% confidence interval 0.01–0.98; *p* = 0.048) was a significant predictor of reduction of limb ischemia.

**Conclusions:**

Simultaneous insertion of a DPC, particularly under fluoroscopy guidance, can be considered as a preventive strategy for limb ischemia in femorally cannulated patients on VA-ECMO.

## Background

Venoarterial extracorporeal membrane oxygenation (VA-ECMO) has been widely used as a salvage therapy in critically ill patients with refractory cardiogenic shock [1]. The femoral vessel approach for VA-ECMO insertion is regarded as the default route because the equipment can be placed rapidly and easily [2]. In patients undergoing VA-ECMO using transfemoral cannulation, limb ischemia is a lethal complication that can be influenced by vessel size, increased vascular tone due to hemodynamic instability, size of the indwelling arterial cannula, and use of vasopressors [3–6]. To prevent limb ischemia after cannulation, the guidance of an antegrade distal perfusion catheter (DPC) into the proximal superficial femoral artery (SFA) has been assisted by various techniques such as ultrasound and fluoroscopy [2, 7]. However, the optimal timing of and strategy for DPC insertion have not been fully elucidated for patients undergoing VA-ECMO via femoral cannulation. Therefore, we investigated whether simultaneous insertion of a DPC, particularly fluoroscopy-guided DPC insertion, at the time of primary ECMO cannulation can reduce critical limb ischemia compared with a provisional approach.

## Methods

### Study population

**Fig. 1:**
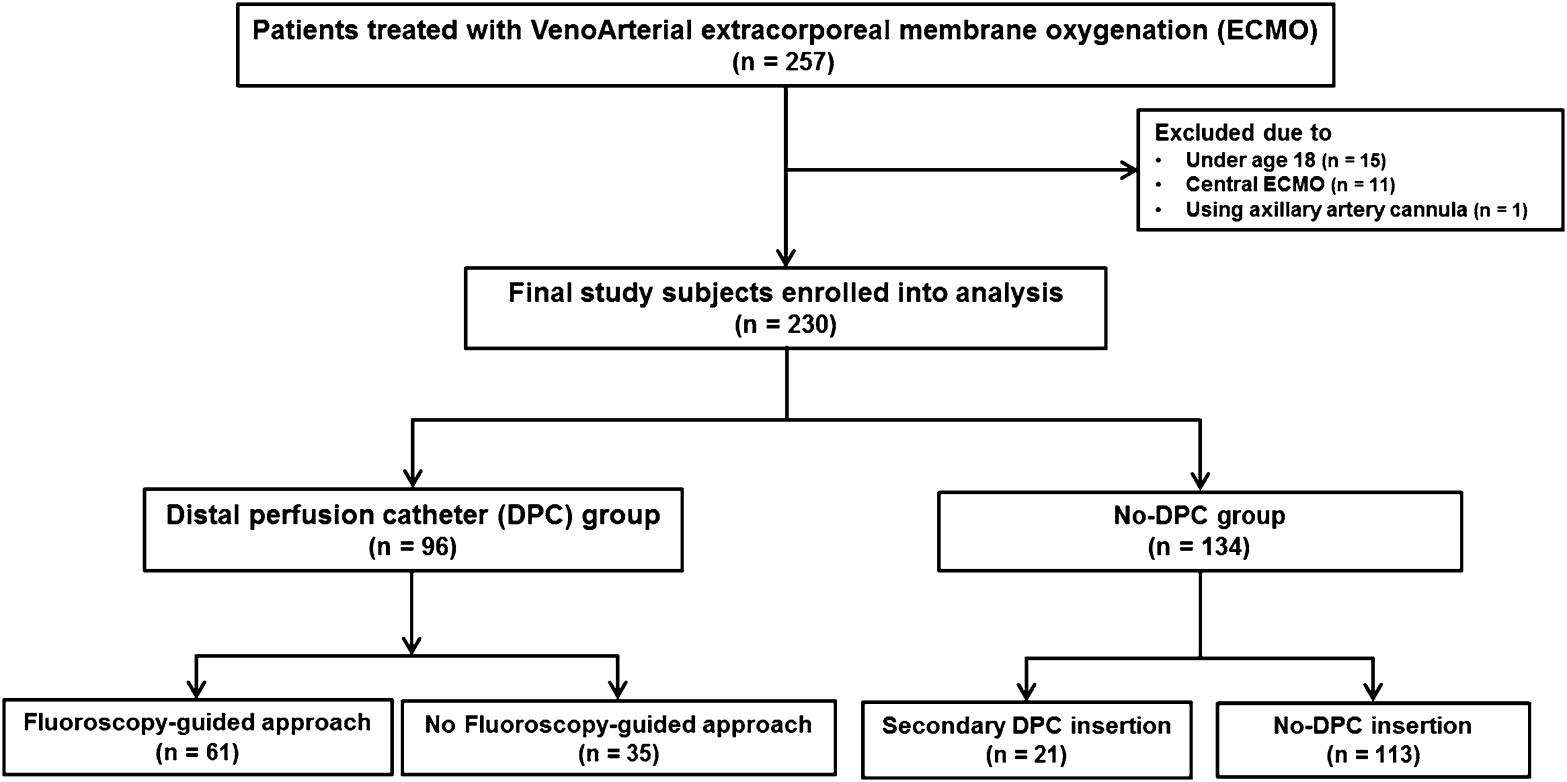
Schematic illustration of study cohort selection

We investigated 257 consecutive patients who underwent VA-ECMO from a retrospective multicenter registry at Samsung Medical Center, Seoul, South Korea, and Samsung Changwon Hospital, Gyeongnam, South Korea, from August 2014 through July 2017. Of these, we included only patients who were placed on peripheral VA-ECMO via femoral cannulation and excluded patients who were under 18 years of age or who underwent ECMO using central aortic or axilla-arterial cannulation. Ultimately, 230 patients were enrolled in this study and were divided into two groups according to timing of DPC insertion: patients who underwent DPC insertion at the time of the primary cannulation (DPC group) and patients who did not undergo DPC insertion at the primary femoral cannulation including provisional DPC insertion after the onset of distal limb ischemia (No-DPC group) (Fig. 1). The local institutional review board of each participating hospital approved this study and waived the requirement for informed consent.

### Extracorporeal membrane oxygenation implantation and management

The decision to implant ECMO was made by an experienced team, and the ECMO was placed by either cardiovascular surgeons or interventional cardiologists. The Capiox Emergency Bypass System (Capiox EBS™; Terumo, Inc., Tokyo, Japan) and Permanent Life Support (PLS) System (MAQUET, Rastatt, Germany) were used. Heparin was intravenously administered as a bolus of 5000 units, followed by continuous intravenous infusion to maintain an activated clotting time between 150 and 180 s. After initiation of ECMO, the pump blood flow rate was initially set above 2.2 L/min/body surface area (m^2^) and subsequently adjusted to maintain a mean arterial pressure above 65 mmHg. Blood pressure was continuously monitored through an arterial catheter, and arterial blood gas analysis was performed in the artery of the right arm to estimate cerebral oxygenation. Additional fluids, blood transfusion, and/or catecholamines (i.e., norepinephrine, epinephrine, or dobutamine) were supplied to maintain intravascular volume and/or to achieve a mean arterial pressure above 65 mmHg if necessary [8].

### Cannulation of extracorporeal membrane oxygenation and distal perfusion catheter

**Fig. 2:**
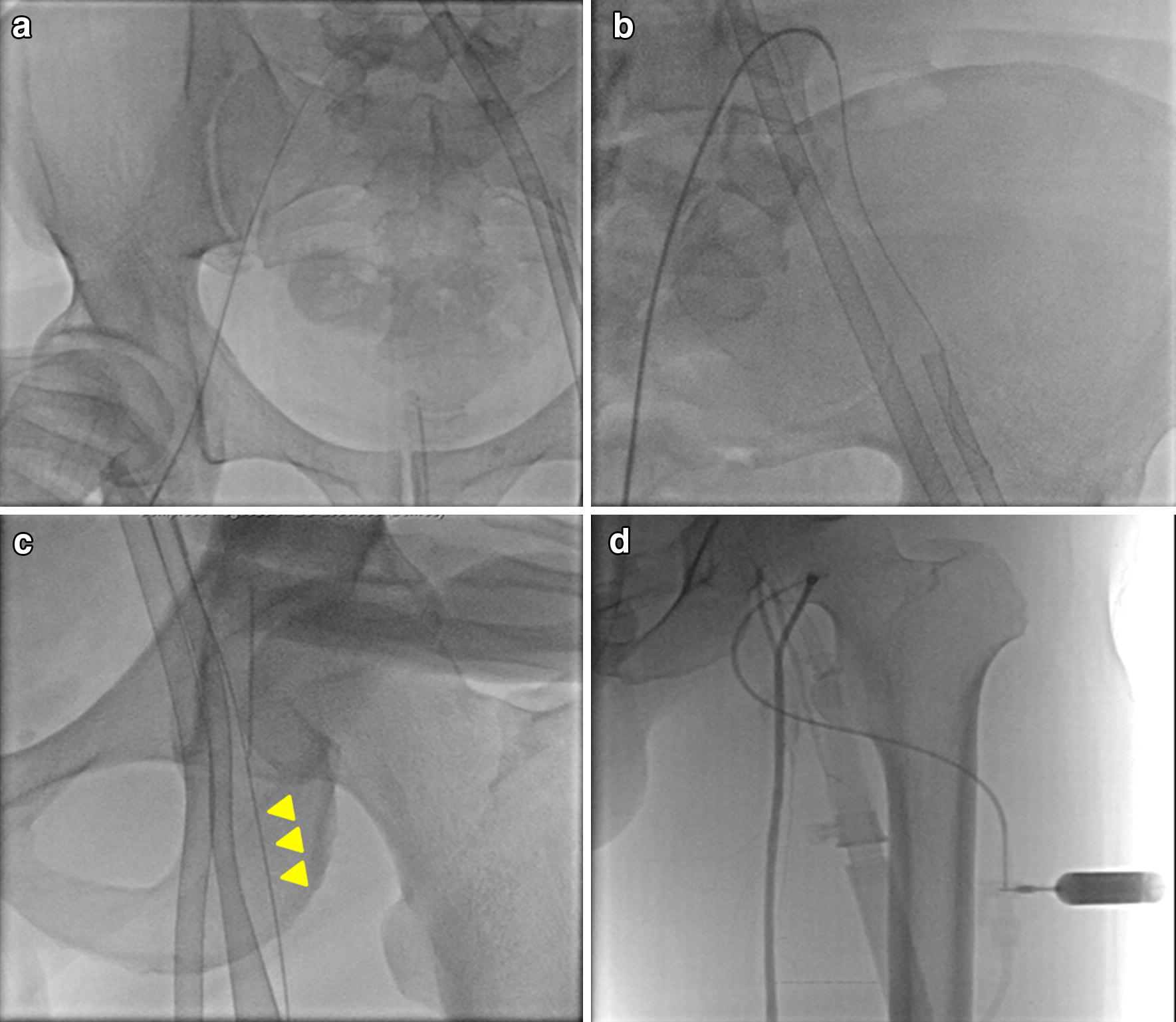
Percutaneous insertion of a distal perfusion catheter. **a** Under fluoroscopy guidance, a sheath at the contralateral common femoral artery (CFA) was inserted using a micropuncture needle followed by a 0.018-inch nitinol wire. **b** A hydrophilic wire was advanced from the sheath of the contralateral CFA (through the aortic bifurcation and the ipsilateral common iliac artery, between the arterial ECMO cannula and the vessel wall of the ipsilateral common iliac artery) to the distal portion of the ipsilateral superficial femoral artery (SFA). **c** The proximal SFA ipsilateral to the arterial cannula was punctured using a micropuncture needle followed by a 0.018-inch nitinol wire as the reference point of the previously placed wire (yellow arrow heads). A distal perfusion catheter (6- or 7-Fr. sheath) was inserted antegrade and advanced safely into the mid-SFA. **d** The distal perfusion catheter was attached to the side port of the arterial cannula using 6-inch extension tubing with an intervening three-way stopcock

Percutaneous cannulation of the femoral artery and vein was mainly performed by the attending staff interventional cardiologist or cardiovascular surgeon using the Seldinger technique. The femoral vessels (either unilateral, one-side arterial, or one-side venous) were accessed retrograde using an angiogram needle. The venous cannula was either 55 or 68 cm in length and from 21 to 28 Fr.; the arterial cannula was 24 cm and from 14 to 21 Fr. The final selection of cannula was based on manufacturer pressure–flow curves for each cannula size and patient size. Femoral cut-down procedures were performed when it was difficult to puncture the femoral artery percutaneously, for example, in patients with peripheral artery disease or severe obesity. At bedside, the DPC placement site was accessed antegrade using a micropuncture needle followed by a 0.018-inch nitinol wire (Cook Medical Inc, Bloomington, IN, USA) at the proximal SFA ipsilateral to the arterial cannula. A 6- or 7-Fr. sheath was then advanced into the mid-SFA. In the catheterization laboratory, we first inserted another sheath at the common femoral artery (CFA) contralateral to the arterial ECMO cannula and advanced a hydrophilic wire from the CFA sheath (through the aortic bifurcation and the ipsilateral common iliac artery, then between the arterial ECMO cannula and the vessel wall of the ipsilateral common iliac artery) to the distal portion of the ipsilateral SFA. The DPC was then safely inserted into the distal portion of the arterial cannula (ipsilateral to the SFA) using a micropuncture needle as the reference point of the previously placed hydrophilic wire. The catheter was attached to the side port of the arterial cannula using 6-inch extension tubing with an intervening three-way stopcock (Fig. 2).

### Data collection, definitions, and study outcomes

Baseline characteristics, procedural characteristics, laboratory data, and clinical outcome data were obtained by reviewing medical records or by telephone contact, if necessary. Laboratory findings, including creatinine and lactate, were collected just before VA-ECMO insertion. The primary outcome was limb ischemia, which was defined as cases requiring surgical management or involving neurologic sequelae. In-hospital mortality, successful weaning rate of ECMO, thrombotic events, major bleeding, and catheter-related complications (defined as a composite of limb ischemia, major bleeding, and thrombotic events) were assessed in addition to the primary outcome. Major bleeding was defined as cases involving hemodynamic instability or those that occurred in a critical area or organ such as intracranial, retroperitoneal, pericardial, or intramuscular with compartment syndrome.

### Statistical analysis

Continuous variables were compared using Student’s *t* test or the Wilcoxon rank-sum test. The results were presented as mean ± standard deviation or median with interquartile range. Categorical data were tested using Fisher’s exact test or the Chi-square test. Multivariable logistic regression analysis was performed via a stepwise backward selection process to determine the independent predictors of limb ischemia. Clinical variables (i.e., fluoroscopy-guided simultaneous DPC, age ≥ 65 years, gender, duration of ECMO > 5 days, and large arterial cannula) were included in the regression models. All variables associated with limb ischemia were analyzed using univariate analysis. Factors with *p* < 0.2 and those that were clinically relevant were included in the multivariable analysis. All tests were two-tailed, and *p* value < 0.05 was considered statistically significant. Statistical analyses were performed using SPSS software, version 23 (IBM, Armonk, New York, USA).

## Results

### Baseline, procedural, and laboratory characteristics

The baseline and procedural characteristics of the study population are shown in Table 1. There were no significant differences between the DPC group and the No-DPC group except body mass index (BMI) in baseline characteristics. Of the 134 patients in the No-DPC group, 21 (15.7%) underwent secondary DPC insertion. ECMO was mainly inserted in either catheterization laboratory room, intensive care unit, or emergency room. Extracorporeal cardiopulmonary resuscitation (ECPR) was more frequently performed in the No-DPC group than in the DPC group (*p* = 0.003). The size of femoral arterial cannula was similar between the DPC group and the No-DPC group (*p* = 0.080), but venous cannular size was larger in the DPC group than in the No-DPC group (*p* = 0.019). Anticoagulation therapy (*p* = 0.004) and left ventricular venting during ECMO support (*p* = 0.029) were more frequently performed in the DPC group than in the No-DPC group, and large arterial cannula (*p* = 0.066) tended to be used less frequently in the DPC group compared to the No-DPC group. The median duration of ECMO support was 3 days [interquartile range (IQR) 1–7 days]. The median total length of stay in the intensive care unit was 6 days (IQR 1–16 days), and the median total length of stay in the hospital was 20 days (IQR 6–45 days).

**Table 1:** Baseline and procedural characteristics

	DPC group (*n* = 96)			No-DPC group (*n* = 134)	*p* value DPC versus No-DPC
	Fluoroscopy-guided (*n* = 61)	No fluoroscopy-guided (*n* = 35)	*p* value		
Age (years)	55.2 ± 16.7	55.7 ± 16.2	0.143	58.5 ± 13.7	0.106
Gender (male)	39 (63.9)	20 (57.1)	0.735	89 (66.4)	0.439
Body mass index (kg/m^2^)	23.0 ± 3.1	25.0 ± 3.9	< 0.001	25.6 ± 3.9	< 0.001
Diabetes mellitus	16 (26.2)	10 (28.6)	0.260	46 (34.3)	0.243
Hypertension	22 (36.1)	10 (28.6)	0.790	51 (38.1)	0.462
Dyslipidemia	11 (18.0)	2 (5.7)	0.016	9 (6.7)	0.083
Current smoker	13 (21.3)	12 (34.3)	0.959	29 (21.6)	0.438
Chronic kidney disease	4 (6.6)	2 (5.7)	0.688	11 (8.2)	0.576
Peripheral vascular disease	2 (3.3)	0 (0)	0.670	3 (2.2)	0.936
Previous MI	10 (16.4)	7 (20.0)	0.396	29 (21.6)	0.462
Previous PCI	9 (14.8)	7 (20.0)	0.217	30 (22.4)	0.285
Previous CABG	1 (1.6)	1 (2.9)	0.136	9 (6.7)	0.104
Previous CVA	5 (8.5)	2 (5.7)	0.858	10 (7.5)	0.961
Clinical presentation			0.002		0.061
Ischemic cardiomyopathy	32 (52.5)	10 (28.6)		58 (43.3)	
Non-ischemic cardiomyopathy	13 (21.3)	5 (14.3)		21 (15.7)	
Septic shock	0 (0)	6 (17.1)		4 (3.0)	
Refractory arrhythmia	8 (13.1)	1 (2.9)		4 (3.0)	
Other causes	8 (13.1)	13 (37.1)		47 (35.1)	
Purpose of ECMO implantation			0.035		0.121
Bridge to recovery	49 (80.3)	32 (91.4)		122 (91.0)	
Bridge to transplantation	12 (19.7)	3 (8.6)		12 (9.0)	
ECPR	24 (39.3)	17 (48.6)	0.002	84 (62.7)	0.003
Initial ECMO pump flow (L/min)	3.0 ± 0.8	3.3 ± 0.5	0.722	3.1 ± 1.1	0.729
Operating site of ECMO			< 0.001		< 0.001
Intensive care unit	0 (0)	21 (60.0)		31 (23.1)	
Catheterization laboratory room	56 (91.8)	0 (0)		33 (24.6)	
Emergency room	4 (6.6)	7 (20.0)		27 (20.1)	
Operating room	0 (0)	4 (11.4)		17 (12.7)	
Others	1 (1.6)	3 (8.6)		26 (19.4)	
Arterial catheter size (Fr.)	15.3 ± 0.7	15.4 ± 0.9	0.061	15.3 ± 0.8	0.080
Venous catheter sized (Fr.)	22.4 ± 1.5	21.9 ± 0.7	0.010	21.5 ± 2.4	0.019
Large arterial cannula^a^	10 (16.4)	9 (25.7)	0.055	41 (30.6)	0.066
During ECMO support					
Anticoagulation therapy	52 (85.2)	25 (71.4)	0.001	84 (62.7)	0.004
Left ventricular venting	16 (26.2)	3 (8.6)	0.003	13 (9.7)	0.029
Distal perfusion	61 (100.0)	35 (100.0)	< 0.001	21 (15.7)	< 0.001
Continuous renal replacement therapy	18 (29.5)	16 (45.7)	0.177	53 (39.6)	0.524
Intra-aortic balloon pump	2 (3.3)	1 (2.9)	0.696	6 (4.5)	0.602
Mechanical ventilation	47 (77.0)	27 (77.1)	0.318	94 (70.1)	0.243
Laboratory findings					
Creatinine (mg/dL) (just before ECMO insertion)	1.4 (1.0–1.54)	1.2 (0.8–1.4)	0.391	1.3 (0.9–1.9)	0.779
Lactate (mmol/L) (just before ECMO insertion)	4.9 (2.8–9.2)	5.7 (2.0–10.4)	0.051	5.3 (2.1–10.1)	0.683
Lactate (mmol/L) (24 h after ECMO insertion)	1.9 (1.4–3.1)	2.2 (1.1–4.9)	0.300	2.1 (0.0–3.9)	0.700
Duration of ECMO support (day)	3.4 (2.1–7.5)	4.0 (2.0–6.3)	0.013	2.0 (1.0–5.0)	0.052
Length of ICU stay (day)	10.0 (3.5–19.0)	6.0 (1.0–15.5)	0.518	5.0 (1.0–10.0)	0.012
Length of hospital stay (day)	27.0 (14.0–73.0)	24.0 (7.5–49.8)	0.271	14.0 (5.0–34.0)	0.030

Values are mean ± standard deviation, median (interquartile range), or n (%)

*CABG* coronary artery bypass grafting, *CVA* cerebrovascular accident, *DPC* distal perfusion catheter, *ECPR* extracorporeal cardiopulmonary resuscitation, *ICU* intensive care unit, *MI* myocardial infarction, *PCI* percutaneous coronary intervention, *ECMO* extracorporeal membrane oxygenation

^a^We considered patient used 16–21-Fr. catheter as patient used large arterial catheter

### Limb ischemia and other catheter-related complications in VA-ECMO patients

Thirty-four cases of ischemic complication (13 limb ischemia, 18 thrombotic events, and 3 ischemic strokes) occurred. Of the 13 patients with distal limb ischemia, 3 were recovered through medical treatment, while 3 underwent fasciotomy, 3 received surgical thrombectomy, and 4 underwent surgical amputation of the distal lower limb implanted with ECMO. Limb ischemia was less frequent in the DPC group than in the No-DPC group (2.1% vs. 8.2%; *p* = 0.047). The incidences of major bleeding (8.3% vs. 4.5%; *p* = 0.228), thrombotic events (5.2% vs. 9.7%; *p* = 0.211), and catheter-related complications (24.0% vs. 19.4%; *p* = 0.405) were not different between the two groups. The rate of successful ECMO weaning was greater in the DPC group than in the No-DPC group (79.2% vs. 61.9%; *p* = 0.005), and the DPC group had a lower tendency of in-hospital mortality than the No-DPC group (38.5% vs. 50.7%; *p* = 0.067) (Table 2).

**Table 2:** Clinical outcomes and complications

	DPC group (*n* = 96)			No-DPC group (*n* = 134)	*p* value DPC versus No-DPC
	Fluoroscopy-guided (*n* = 61)	No fluoroscopy-guided (*n* = 35)	*p* value		
Limb ischemia	1 (1.6)	1 (2.9)	0.688	11 (8.2)	0.047
Major bleeding	5 (8.2)	3 (8.6)	0.949	6 (4.5)	0.228
Thrombotic event	2 (3.3)	3 (8.6)	0.261	13 (9.7)	0.211
Catheter-related complication^a^	12 (19.7)	11 (31.4)	0.194	26 (19.4)	0.405
Successful weaning from ECMO	49 (80.3)	27 (77.1)	0.711	83 (61.9)	0.005
In-hospital mortality	22 (36.1)	15 (42.9)	0.510	68 (50.7)	0.067

Values are *n* (%)

*DPC* distal perfusion catheter, *ECMO* extracorporeal membrane oxygenation

^a^Catheter-related complication was defined as a composite of limb ischemia, cannular site bleeding, and thrombotic event

### Fluoroscopy-guided distal perfusion and predictors on lower limb ischemia

Of the 96 patients in the DPC group, 61 (63.5%) underwent DPC insertion under fluoroscopic guidance (Table 1). Fluoroscopy-guided DPC group had a numerically low incidence of catheter-related complication including limb ischemia, cannular site bleeding, and thrombotic event compared to no fluoroscopy-guided DPC group (Table 2). Furthermore, the incidence of limb ischemia tended to be lower in the fluoroscopy-guided DPC group than in the No-DPC group (*p* = 0.057). We performed multivariable logistic regression analysis to identify predictors of limb ischemia in patients undergoing VA-ECMO. Simultaneous DPC insertion (OR 0.13, 95% CI 0.03–0.68, *p* = 0.016) and ICU stay ≥ 11 days (OR 4.34, 95% CI 1.26–14.97, *p* = 0.020) on model I, and fluoroscopy-guided simultaneous DPC insertion (OR 0.11, 95% CI 0.01–0.98, *p* = 0.048) and ICU stay ≥ 11 days (OR 3.71, 95% CI 1.12–12.32, *p* = 0.032) on model II were significant prognostic factors for lower limb ischemia (Table 3).

**Table 3:** Predictors of lower limb ischemia

	Odds ratio	95% CI	*p* value
Model I			
Simultaneous DPC insertion	0.13	0.03–0.68	0.016
Age ≥ 65 years	0.27	0.06–1.35	0.111
Male	1.37	0.40–4.76	0.618
BMI ≥ 25 kg/m^2^	0.46	0.13–1.66	0.235
Diabetes mellitus	0.91	0.25–3.35	0.891
Clinical presentation	0.77	0.52–1.14	0.189
ICU stay ≥ 11 days	4.34	1.26–14.97	0.020
Model II			
Fluoroscopy-guided DPC insertion	0.11	0.01–0.98	0.048
Age ≥ 65 years	0.28	0.06–1.36	0.114
Male	1.46	0.42–5.10	0.554
BMI ≥ 25 kg/m^2^	0.48	0.14–1.70	0.255
Diabetes mellitus	0.93	0.25–3.45	0.910
Clinical presentation	0.73	0.49–1.10	0.131
ICU stay ≥ 11 days	3.71	1.12–12.32	0.032

Model I: adjusted with simultaneous DPC insertion, age ≥ 65 years, male, BMI ≥ 25 kg/m^2^, diabetes mellitus, clinical presentation, and ICU stay ≥ 11 days

Model II: adjusted with fluoroscopy-guided simultaneous DPC insertion, age ≥ 65 years, male, BMI ≥ 25 kg/m^2^, diabetes mellitus, clinical presentation, and ICU stay ≥ 11 days

*BMI* body mass index, *CI* confidence interval, *DPC* distal perfusion catheter, *ICU* intensive care unit

## Discussion

We investigated the association between the method and timing of distal perfusion and vascular complications, including limb ischemia, major bleeding, and thrombotic events, in patients undergoing VA-ECMO. Our main finding is that simultaneous DPC insertion at the time of primary ECMO cannulation reduced the incidence of lower limb ischemia. In the multivariable analysis, fluoroscopy-guided simultaneous insertion of DPC, duration of ECMO implantation > 5 days, and use of a large arterial cannula (over 16 Fr.) were significant predictors of limb ischemia. In general, our findings correspond well with earlier studies that established an association between distal perfusion and adverse clinical outcomes [2, 9]. The present study showed for the first time that fluoroscopy-guided DPC insertion via a contralateral approach might be a safe and effective strategy to prevent limb ischemia in femorally cannulated patients on VA-ECMO.

VA-ECMO implantation for patients with refractory cardiopulmonary failure is quick and convenient when using a percutaneous femoral approach, but limb ischemia and other catheter-related complications frequently develop due to partial luminal obstruction or injury to the common femoral artery or vein. Muehrcke et al. [10] reported an ischemia rate of 70% in an ECMO population of 24 patients without DPC placement at the time of cannulation. Their cannulation protocol was modified to include simultaneous DPC placement, with noted improvement in limb salvage. Foley et al. [5] reported an ischemia rate of 21% in 58 patients without DPCs, although they found no difference in limb ischemia or mortality between prophylactic and expectant placement of a DPC. These findings strongly suggest that limb ischemia can be avoided in a large number of patients undergoing ECMO if the physician can safely insert a DPC. Additionally, Lamb et al. [2] reported that placement of a DPC at the time of cannulation and intensive monitoring of limb perfusion may decrease the incidence of ischemic complication. Ranney et al. [9] investigated the indication and timing of DPC placement and reported that DPC placement at the time of primary cannulation may lower the incidence of limb ischemia. However, both studies had a limited number of patients and focused only on the occurrence of complicated limb ischemia in ECMO patients; therefore, no definite conclusion can be drawn from these studies. The strength of our study is in the comparison of overall clinical outcomes and ECMO-related vascular complications, as well as limb ischemia, between two timings of DPC insertion using a large, dedicated ECMO registry.

In the real-world practice, additional procedures, like DPC insertion, could be harmful in critically ill patients who are vulnerable to bleeding or who had received anticoagulation therapy while on ECMO. Therefore, finding a method to insert the DPC that avoids bleeding complications caused by multiple needle thrusts would make it possible to avoid fatal complications in the lower limb. In the present study, DPCs were preferentially inserted in all VA-ECMO patients unless limited by technical considerations, typically an inability to cannulate the SFA, and no procedural-related limb complication occurred in patients treated with fluoroscopy-guided DPC insertion. Our findings suggest that simultaneous DPC insertion at the time of primary ECMO cannulation should be considered to prevent limb ischemia, and image-guided insertion methods, such as fluoroscopy, are more effective and safe.

### Study limitations

This study has several limitations. First, its design was non-randomized, retrospective, and observational, which may have affected the results due to confounding factors. Second, the selection of treatment strategy for the cannulated limb was at the discretion of the operator and could have influenced the results by introducing bias. Third, the impact of limb ischemia on long-term outcomes and the influence of eventual distal perfusion in the No-DPC group were not assessed in this study. Fourth, patients undergoing simultaneous DPC insertion may be comparatively more stable to those who do not undergo the procedure. The DPC and No-DPC groups were different in baseline BMI, rate of ECPR, and operating site of ECMO in the present study. This might have influenced study outcomes and produced a higher rate of ECMO weaning in patients undergoing simultaneous DPC insertion. Finally, we did not compare our fluoroscopy-guided contralateral DPC strategy with previous methods such as ultrasound-guided DPC insertion or ipsilateral DPC insertion. Therefore, this method cannot be chosen with certainty as the best method of DPC insertion.

## Conclusion

Simultaneous insertion of DPC at the time of primary ECMO cannulation using a femoral approach could prevent advanced limb ischemia. In particular, fluoroscopy-guided DPC insertion via a contralateral approach can be considered as a new strategy for prevention of disastrous vascular complications.

## Abbreviations

DPC: distal perfusion catheter
SFA: superficial femoral artery
VA-ECMO: venoarterial extracorporeal membrane oxygenation
